# DNA methylome profiling of all-cause mortality in comparison with age-associated methylation patterns

**DOI:** 10.1186/s13148-019-0622-4

**Published:** 2019-02-08

**Authors:** Jesper Beltoft Lund, Shuxia Li, Jan Baumbach, Anne Marie Svane, Jacob Hjelmborg, Lene Christiansen, Kaare Christensen, Paul Redmond, Riccardo E. Marioni, Ian J. Deary, Qihua Tan

**Affiliations:** 1 Epidemiology and Biostatistics, Department of Public Health, Faculty of Health Science, University of Southern Denmark, J. B. Winsløws Vej 9B, DK-5000 Odense, Denmark; 2 Unit of Human Genetics, Department of Clinical Research, University of Southern Denmark, Odense, Denmark; 3 Department of Mathematics and Computer Science, University of Southern Denmark, Odense, Denmark; 4 Chair of Experimental Bioinformatics, School of Life Sciences Weihenstephan, Technical University of Munich, Munich, Germany; 5 Department of Psychology, University of Edinburgh, Edinburgh, Scotland UK; 6 Center for Genomic and Experimental Medicine, University of Edinburgh, Edinburgh, Scotland UK; 7 Center for Cognitive Aging and Cognitive Epidemiology, University of Edinburgh, Edinburgh, Scotland UK

**Keywords:** Mortality, Epigenome-wide association study, Aging, DNA methylation, Old cohorts

## Abstract

**Background:**

Multiple epigenome-wide association studies have been performed to identify DNA methylation patterns regulated by aging or correlated with risk of death. However, the inter-relatedness of the epigenetic basis of aging and mortality has not been well investigated.

**Methods:**

Using genome-wide DNA methylation data from the Lothian Birth Cohorts, we conducted a genome-wide association analysis of all-cause mortality and compared this with age-associated methylation patterns reported on the same samples.

**Results:**

Survival analysis using the Cox regression model identified 2552 CpG sites with genome-wide significance (false discovery rate < 0.05) for all-cause mortality. CpGs whose methylation levels are associated with increased mortality appear more distributed from the gene body to the intergenic regions whereas CpGs whose methylation levels are associated with decreased mortality is more concentrated at the promoter regions. In comparison with reported CpGs displaying significant age-dependent methylation patterns in the same samples, we observed a limited but highly significant overlap between mortality-associated and age-associated CpGs (*p* value 2.52e−06). Most importantly, the overlapping CpGs are dominated by those whose overall age-related methylation patterns reduce the risk of death.

**Conclusion:**

All-cause mortality is significantly associated with altered methylation at multiple genomic sites with differential distribution in gene regions for CpGs correlated with increased or decreased risk of death. The age-dependent methylation changes could reflect an active response to the aging process that contributes to maintain individual survival.

**Electronic supplementary material:**

The online version of this article (10.1186/s13148-019-0622-4) contains supplementary material, which is available to authorized users.

## Background

As an important mechanism in epigenetic regulation, DNA methylation (DNAm) has been shown to have significant impacts on biological processes (aging, development) and diseases [1]. Recent advancement in genomic analysis has enabled collection of large-scale DNAm data at genome levels on samples from older-aged individuals, such as the Lothian Birth Cohort (LBC) study [2], for global profiling of the aging DNA methylome. For example, a recent epigenome-wide association study (EWAS) identified a large number of 67,604 differentially methylated genomic sites associated with age in the LBC samples (family-wise error rate, FWER < 0.05) of which 5168 sites were replicated in independent samples [3].

Although studying the age-related change in DNAm can help to assess the epigenetic regulation during human aging [4–6] and might be relevant to age-related changes in health [7], it would be valuable to examine directly the epigenetic associations with death or all-cause mortality, an objective measure of the overall health of a population. In the literature, several EWASs on all-cause mortality have been published, for example, by Zhang et al. [8] and by Svane et al. [9], revealing significant sites in association with overall risk of death although with low or even no overlap. Because current EWAS has been conducted either on chronological age or mortality, the epigenetic inter->relationship between aging and mortality has not been well described.

Using the LBC data, we performed survival analysis on genome-wide DNA methylation levels with two aims: first, to look for epigenetic markers of all-cause mortality in the older-aged LBC cohorts, and second, to explore the epigenetic link between all-cause mortality and the aging process, by comparing mortality-associated methylation changes with age-associated methylation patterns. The second aim took advantage of the published EWAS on age-related changes in DNA methylation levels in the same LBC samples from Li et al. [3].

## Methods

### The Lothian Birth Cohort samples

The LBC samples consist of 2195 blood samples taken from 1425 individuals of two birth cohorts born in 1921 and 1936 (Table 1), with between 1 and 4 whole blood samples per participant collected over the follow-up waves.

**Table 1:** The Lothian Birth Cohorts LBC1921 and LBC1936 with elderly participants conducted from 1999 and onward

Dataset	LBC1921	LBC1936	Total
*N*	476	949	1425
Women	286 (60%)	471 (50%)	757
Deaths	365 (77%)	154 (16%)	519
Age (years) at blood sampling (mean)	78–95 (79)	67–77 (70)	67–95
Year(s) of blood sampling	1999–2013	2004–present	

The 1921 birth cohort (LBC1921) was collected in the period 1999–2013. The mean initial recruitment age was 79 years, with 550 individuals (476 with methylation data) at the start. The 1936 birth cohort (LBC1936) was collected from 2004 to date and contains 1091 individuals (949 with methylation data) with a mean age at initial recruitment of 70 years. Both are longitudinal samples with repeated visits about every 3 years in the Lothian region (Edinburgh and its surrounding areas) of Scotland. The LBC samples have been described in open-access protocol and profile articles [2, 10, 11]. During each wave, the blood sample for each participant was collected. Genome-wide DNA methylation was analyzed using the Illumina Human Methylation 450K Beadchip containing 485,512 CpG sites. For this study, only the DNA methylation data from the last available blood sample of each individual were analyzed for association with mortality. The LBC data are accessible through the European Genome-phenome Archive (https://www.ebi.ac.uk/ega/home) with an accession number EGAS00001000910.

Ethics permission for the LBC1921 was obtained from the Lothian Research Ethics Committee (Wave 1: LREC/1998/4/183). Ethics permission for the LBC1936 was obtained from the Multi-Center Research Ethics Committee for Scotland (Wave 1: MREC/01/0/56), the Lothian Research Ethics Committee (Wave 1: LREC/2003/2/29). Written informed consent was obtained from all subjects. The study was conducted in accordance with the principles of the Helsinki Declaration.

### DNA methylation data pre-processing

We removed polymeric probes with European allele frequency above 1% (10,627 CpGs), cross-reactive probes (29,233 CpGs) [12], and CpGs with more than 5% missing values or detection *p* values > 0.05 across all samples (89 CpGs) as probe-level quality control (QC). CpGs from the sex-chromosomes (X and Y) were also removed from the analysis (11,648 CpGs). After preprocessing, 445,544 out of the 485,512 CpGs remained. Sample level QC was performed using conventional quality control measurements by removing samples in which > 1% of the probes had a detection *p* value > 0.05, estimated using the R package *minfi* [13]. For subsequent analysis, the methylation *β* values were logit transformed into methylation *M* value as *M* = log_2_ (*β*/(1−*β*)).

To account for cell composition effect when measuring DNA methylation in whole blood samples, blood cell-type composition has been estimated using Houseman’s method [14] implemented in the R package *celltypes450* (https://github.com/brentp/celltypes450). The package estimated cell-type proportions for CD8T, CD4T, natural killer cell (NK), B cell, monocyte, and granulocyte which were included in regression models as covariates for adjusting blood cell heterogeneity.

To control for potential batch effect, we performed a principal component analysis (PCA) on the cohorts and found a subgroup belonging to a specific line of Illumina 450K BeadChip batches (*N* = 129) (Additional file 1: Figure S1). Based on the top 2 principal components from PCA, we ran ComBat [15] using the R package *sva* [16] to remove the batch effect. As shown by Additional file 1: Figure S1, ComBat successfully removed the batch effect. Correction for batch effect enabled joint analysis of the 1921 and 1936 birth cohorts, providing higher statistical power for the EWAS.

### Statistical analysis

We fitted a Cox regression model for each CpG to estimate the association between methylation and mortality adjusting for age at blood sampling, sex, and correcting for cell composition. Time-to-event was defined as Δage, measuring the time in years from blood sampling to death (with right-censoring). Correcting for multiple testing was done by calculating the false discovery rate (FDR) [17] and defining FDR < 0.05 as genome-wide significance. R scripts for data analysis including data preprocessing have been deposited in GitHub at https://github.com/Silver-Hawk/Lund_et_al_2019_EWAS_AllCauseMortalityAgePatterns

### Pathway analysis

The identified CpGs were linked to nearest genes using the Illumina 450K BeadChip annotation file and split into two sets with either negative or positive mortality associations. Each set was tested for over-representation of gene sets (pathways) using the Molecular Signatures Database through Gene Set Enrichment Analysis (GSEA) [18] based on the canonical pathway’s curated gene sets (*CP*, 1329 gene sets in the collection). The over-representation analysis is a statistical test based on hypergeometric distribution for testing if the submitted list of genes contains more genes from a pathway or gene set than would be expected by chance (see below). The test produces a probability score for each pathway or gene set, which is corrected by calculating FDR using the Benjamini-Hochberg method [17].

### Hypergeometric test

We used a hypergeometric test both for pathway analysis as described above and for testing if the age-associated CpGs (*N* = 67,604) from Li et al. [3] are over-represented in the significant CpGs for mortality. Assuming *N* is the total number of CpGs on the 450K array after QC (here, *N =* 445,544 CpGs), *m* the number of significant CpGs for mortality, *n* the number of CpGs significantly regulated by age, and *k* the number of overlapping CpGs significant for both age and mortality. The probability for randomly observing *X ≥ k* age-regulated CpGs in the *m* mortality related CpGs can be calculated as:

$$ p\left(X\ge k\right)=1-{\sum}_{r=0}^k\left(\frac{m}{r}\right)\left(\frac{N-m}{n-r}\right)/\left(\frac{N}{n}\right). $$

For pathway over-representation analysis, *N* is the number of genes linked to all CpGs on the 450K array, *m* is the number of genes linked to mortality-related CpGs, *n* is the number of genes in a particular biological pathway, and *k* is the number of genes belonging to both the pathway under testing and the list of genes linked to mortality-related CpGs.

## Results

### Single CpG-based EWAS

**Fig. 1:**
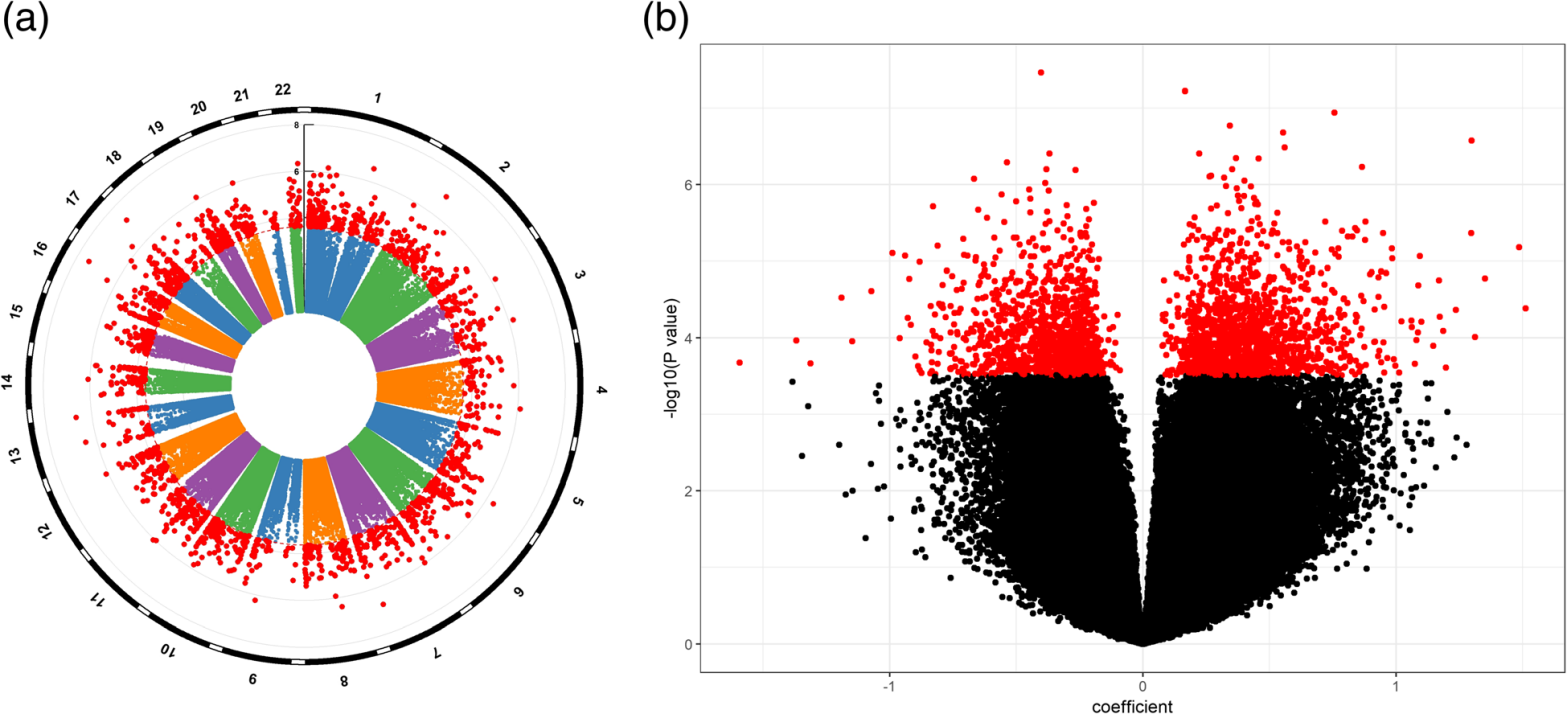
Illustration of results from EWAS on mortality presented by plotting − log_10_(*p* value) against CpG’s chromosomal location (**a**, Manhattan plot) and CpG’s regression coefficient in the Cox model (**b**, volcano plot)

Applying the Cox regression model to each of the measured CpGs on the LBC samples (*N* = 1425, deaths = 519) identified a total of 2552 CpGs significantly associated with mortality, with FDR < 0.05. Distributions of chromosome-wise and overall *p* values are depicted in Fig. 1a and b; CpGs with FDR < 0.05 are marked in red. Of these, 1403 were positively associated (Fig. 1b, right) and 1149 negatively associated (Fig. 1b, left) with mortality. From Fig. 1b, we see that there are more CpGs whose methylation levels are positively correlated with mortality than CpGs displaying negative correlation. Table 2 shows 22 CpGs with *p* value < 1e−06. Among them, only 7 are negatively associated with mortality. The full list of significant CpGs (FDR < 0.05) with their associated genes is reported in Additional file 2: Table S1. The QQ plot (Additional file 3: Figure S2) indicates a large number of CpGs displaying potential contribution to all-cause mortality but only the top ones reached genome-wide significance (FDR < 0.05, red dots) after correcting for multiple testing.

**Table 2:** Statistics and annotations for the top 22 CpGs with *p* < 1e−06

CpG*	HR^1^	SE	*Z* score	*p* value	FDR*	CHR*	MAPINFO*	Gene*	Gene group*	CpG island*
cg00154443	0.6695028	0.072733	− 5.51634	3.46E−08	0.008403	16	71917454	ATXN1L;ZNF821	Body;TSS200	N_Shore
cg03636200	1.1806664	0.030655	5.417627	6.04E−08	0.009774	17	80005395			N_Shelf
cg04157272	2.1306391	0.142672	5.301819	1.15E−07	0.013814	2	68017838			
cg03217572	1.4100455	0.065719	5.228685	1.71E−07	0.013814	7	142561918	EPHB6	Body	
cg01083884	1.7394170	0.106624	5.191615	2.08E−07	0.01446	13	113641411	MCF2L	Body	
cg07202479	3.6623902	0.252289	5.145359	2.67E−07	0.014684	1	159174162	DARC	TSS1500	
cg10741568	1.7502471	0.109628	5.105981	3.29E−07	0.014684	13	49546137			N_Shelf
cg27649037	1.2494070	0.043894	5.072916	3.92E−07	0.014684	8	53322510	ST18	TSS200	
cg16473125	0.6918957	0.072615	− 5.07224	3.93E−07	0.014684	16	55090720			Island
cg09049436	1.4448276	0.072928	5.045959	4.51E−07	0.014779	5	178854758			
cg14517004	1.5786783	0.090526	5.043703	4.57E−07	0.014779	22	48946660	FAM19A5	Body	
cg06654691	0.5845868	0.1069	− 5.02195	5.12E−07	0.015521	17	40825980	PLEKHH3	Body	N_Shore
cg06936355	2.3773894	0.173358	4.995453	5.87E−07	0.015694	9	99213110	HABP4	Body	Island
cg24860169	1.4223750	0.070717	4.98224	6.29E−07	0.015694	1	46924753			
cg22819488	0.6837794	0.076321	− 4.98058	6.34E−07	0.015694	3	28390286	AZI2;AZI2	1stExon;5′UTR	Island
cg07552157	0.7664161	0.053454	− 4.97678	6.47E−07	0.015694	3	142720316	SR140	TSS200	Island
cg00003305	1.3086604	0.054424	4.94274	7.70E−07	0.016988	19	30715645			Island
cg04402350	1.3023196	0.053478	4.939353	7.84E−07	0.016988	4	146857565	ZNF827	Body	Island
cg09724798	1.3792984	0.06519	4.932918	8.10E−07	0.016988	12	119741507	LOC144742	TSS1500	
cg16503753	0.5136465	0.135248	− 4.92589	8.40E−07	0.016988	14	64319245	SYNE2	TSS1500	N_Shore
cg01773858	1.4928783	0.081542	4.914106	8.92E−07	0.017321	11	73076892	ARHGEF17	Body	S_Shelf
cg12145550	0.6807161	0.078476	− 4.90104	9.53E−07	0.017801	20	44600942	ZNF335	TSS200	Island

^1^Hazard ratio, compared to baseline hazard

*Fields are gathered from the Illumina 450K BeadChip annotation file

**Fig. 2:**
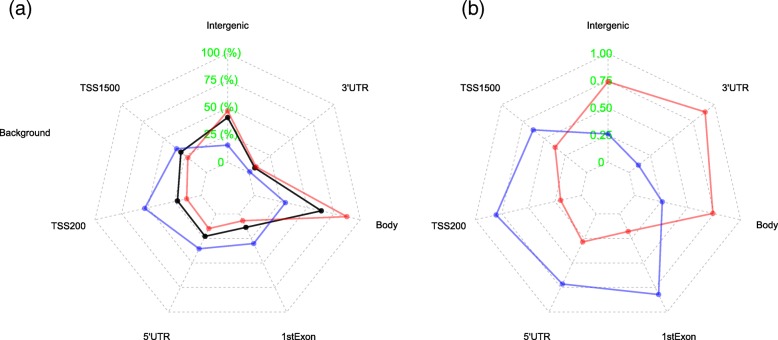
Star plot based on CpGs showing positive (red) and negative (blue) associations with mortality. The arms of the stars represent gene regions where CpGs are located. **a** Proportions of CpGs distributed over different regions for positive (red) and negative (blue) mortality-associated CpGs, as well as for all the CpGs on the array (black). **b** The absolute proportions of positive (red) and negative (blue) mortality-associated CpGs at each gene region

We compared the distribution over gene regions between the two sets of CpGs with positive and negative associations with mortality, to check if there were any differences in their genomic locations (Fig. 2). CpGs associated with increased mortality when methylated (red) appear noticeable more distributed from the gene body to the intergenic regions whereas CpGs associated with decreased mortality (blue) are more concentrated at the promoter regions, in comparison with the distribution of all CpGs on the 450K array (black) in Fig. 2a. A more striking picture is shown by Fig. 2b which displays the frequency of positive (red) and negative (blue) mortality association CpGs in all mortality-associated CpGs at each region, which shows a striking split in gene regions between the two sets. The frequency in Fig. 2b is an absolute frequency of positive and negative mortality association CpGs calculated at each region while the frequency in Fig. 2a is a relative proportion of positive or negative mortality-associated CpGs over the regions.

### Pathway analysis

As described in the “Methods” section, the CpGs significantly associated with mortality were first linked to their annotated functional genes. Submitting each set of genes showing negative (*N* = 936) and positive (*N* = 1151) association with mortality separately to GSEA identified 175 and 200 pathways significantly enriched by each of the two lists of genes (FDR < 0.05) (Additional file 4: Table S3 and Additional file 5: Table S2). Comparing the pathways in the two tables, there are shared top pathways such as the MAPK signaling pathway. However, the most significant pathways are characterized by those involving immune function for genes linked to negative mortality association CpGs, whereas for positive mortality association CpGs, the most significant pathways are characterized by genes encoding the extracellular matrix (ECM) proteins.

### Epigenetic link between aging and mortality

**Fig. 3:**
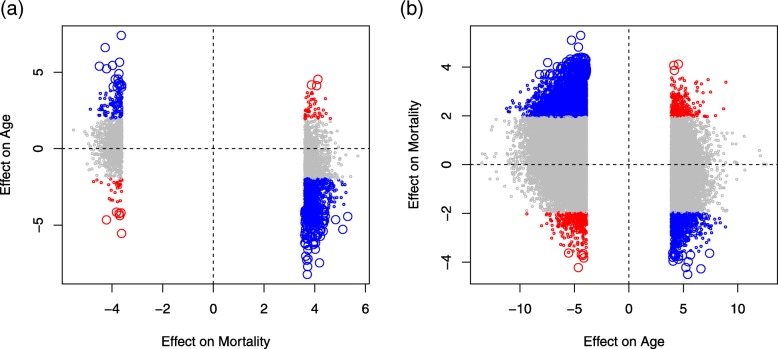
Scatter plots for mortality-associated CpGs plotted against their coefficients for age from the linear model in the EWAS on aging (**a**) and for aging-associated CpGs plotted against their coefficients from the Cox model in the EWAS on mortality (**b**). The colored large dots are 178 CpGs with genome-wide significance in both the EWAS on aging (FWER < 0.05) and the EWAS on mortality (FDR < 0.05)

Using the estimates on age-associated methylation changes from Li et al. [3], we explore the relationship in DNA methylation regulation between mortality and aging. In Fig. 3a, we plot the test statistic (*z* value from Cox regression model) on each of the 2552 significant CpGs associated with mortality (FDR < 0.05) on the *x*-axis against the corresponding test statistic (*t* value from linear regression model) on age (*p* < 0.05 colored in red or blue) on *y*-axis. Among the 1403 CpGs positively associated with mortality (Fig. 3a, right panel), a large number (580 CpGs, 41.3%) have decreased methylation and only a small number (28 CpGs, 2%) has increased methylation with aging. The other half (795 CpGs, 57%) of the positive mortality association CpGs do not show any significant age pattern (*p* > 0.05). Among the 1149 negative mortality association CpGs (Fig. 3a, left panel), 113 (9.8%) CpGs have increased methylation and only 25 (2.2%) CpGs have decreased methylation with aging. Methylation level at the majority (1011 CpGs, 88%) of the negative mortality association CpGs is not affected by aging (*p* > 0.05). In Fig. 3a, CpGs positively associated with mortality but hypermethylated with age or negatively associated with mortality but hypomethylated with age convey increased risk of death as a result of aging and are marked in red; CpGs positively associated with mortality but hypomethylated with age or negatively associated with mortality but hypermethylated with age are beneficial to survival during aging and are marked in blue. Using hypergeometric test, we estimated the probability of observing the number of colored CpGs in each of the four areas in Fig. 3a. Only the blue-colored CpGs in the bottom-right showed a *p* value of 2.52e−06, suggesting it is very unlikely to observe 580 or more CpGs (*k*) demethylated with age (*p* < 0.05) if we randomly take 1403 CpGs (*m*) from all CpGs tested (*N*) among which 158,144 CpGs (*n*) are demethylated with age (*p* < 0.05, EWAS results from Li et al. [3]).

Figure 3b plots the *t* values for the 67,604 age-regulated significant CpGs (FWER < 0.05) from the EWAS on aging by Li et al. [3] on the *X*-axis against their *z*-statistic for mortality association in the Cox models fitted in this study. Of the 9688 CpGs methylated with age (Fig. 3b, right panel), 841 (8.7%) are negatively and 239 (2.5%) are positively associated with mortality (*p* < 0.05), with the majority (88.8%) having no connection with mortality (*p* > 0.05). Of the 57,916 CpGs demethylated with age, 367 (0.6%) are negatively and 6328 (10.9%) are positively associated with mortality; again, the majority (88.5%) are not related to mortality (*p* > 0.05). Similar to Fig. 3a, red/blue colors represent CpGs whose age-associated methylation patterns increase/decrease the risk of death. Applying the hypergeometric test, we found the distributions of blue-colored CpGs in Fig. 3b are significantly different from random with *p* value = 1.88e−61 for the bottom-right area (*n =* 21,281, *m =* 9688, *k =* 841) and *p* = 2.12e−71 for the top-left area (*n =* 39,679, *m =* 57,916, *k =* 6328), suggesting that the probabilities for randomly observing the number or more of the blue CpGs in the two areas are extremely low. Tests for the distribution of the number of red CpGs showed no difference from random.

Finally, Fig. 3 also presents a small group of 178 CpGs with genome-wide significance in both EWAS on mortality (FDR < 0.05) and EWAS on aging (FWER < 0.05) (same set of the large dots in Fig. 3a, b).

## Discussion

Compared with the large number of age-regulated CpGs (*N* = 67,604) found in the LBC cohorts by Li et al. [3], the 2552 CpGs (FDR < 0.05) detected in association with all-cause mortality in this study is in great contrast in magnitude. A large number of age-related CpGs have also been reported by Moor et al. [19] with 56,579 CpGs, of which less than 0.05% were associated with mortality. Although the number of significant sites identified depends largely on the power of a study, the striking difference in the number of significant sites for aging and mortality from the same relatively large LBC sample could indicate that aging as a complex process can involve numerous epigenetic remodeling in the DNA methylome to a larger extent than that for mortality. Note that the above results were all based on blood samples after adjusting for cell compositions. Tan et al. [20] discussed the importance of considering cell type heterogeneity in epigenetic studies on aging using blood samples and different options of adjustment. According to Tan et al. [20], the widely accepted adjustment scheme that controls for cell compositional effect is an efficient handling of the cell type issue in consideration of statistical power and systematic bias [21].

Our findings on the relationship between mortality- and age-associated CpGs seem contradictory to general expectation as aging is related to increased risk of death. The patterns showing in Fig. 3 are based on coefficients from regression models; thus, the conclusions are made on the mean levels. Taking the blue CpGs in the bottom-right panel of Fig. 3a for example, because of their positive effects on mortality, hypermethylation at these CpGs lead to increased risk of death. However, according to their age-dependent methylation patterns, the mean methylation levels decrease with increasing age. This means increased mortality among a group of individuals with increased methylation by age. However, since the mean methylation is going down with age, the effects of age-associated methylation change at these CpGs do not, on average, increase the hazard of death. We postulate that the significantly over-represented protective effects of age-associated methylation patterns could reflect the active response to aging that helps to maintain survival or, in other words, the main stream of age-dependent methylation patterns might represent an epigenetic mechanism for successful aging. The finding merits further investigation with a focus on the causation of DNA methylation and aging to provide clues for elucidating the underlying biology.

As discussed above, the positive and negative mortality association CpGs in Fig. 3 manifest differently in terms of aging. From Fig. 2, we see that they are also differentially distributed over gene regions with positive mortality association CpGs distributed mainly from gene body to the intergenic region and negative mortality association CpGs in the promoter regions. The differential distribution could help to explain the functional differences of the two lists of CpGs. The top biological pathways significantly over-represented by genes linked to the positive mortality association CpGs (Additional file 4: Table S3) are mainly implicated in the function of ECM while the most significant pathways from genes linked to the negative mortality association CpGs (Additional file 5: Table S2) are extensively involved in immune functions. The molecular or biological basis of these differences merits further investigation.

Svane et al. [9] performed an EWAS on mortality in middle-aged and old Danish twins (*N* = 870, deaths = 258) using the same platform for DNA methylation analysis as in our study. A total of 2806 significant CpGs with FDR < 0.05 was revealed. In our primary analysis, without adjusting for the cohort effect between LBC1921 and LBC1936, there were 7 significant CpGs (FDR < 0.05) overlapped with the Danish study. After we corrected the cohort effect using ComBat, no overlapping CpG with FDR < 0.05 was found. There were 57 overlapping CpGs (43 positively and 14 negatively associated with mortality) with *p* < 0.05 in their study. In another EWAS on all-cause mortality, Zhang et al. [8] reported a list of 58 CpGs from blood samples. Again, no overlap was observed with this study. It has already been shown that low or no replication is a frequent phenomenon in (epi)genome-wide association studies [22]. For example, of the 2806 CpGs reported by Svane et al. [9], only 2 overlapped with the CpGs found by Zhang et al. [8] and none with the mortality-related CpGs reported by a Finnish EWAS [23]. Future large-scale consortium studies and meta-analysis should be encouraged to narrow down the epigenetic targets for all-cause mortality.

Considering methylation changes at different CpGs in the same regulatory region or gene body could alter the expression of the same gene, we additionally performed gene-level replication using the EWAS results from Danish twins. Among their 2806 significant CpGs with FDR < 0.05, 2112 CpGs could be linked to nearby genes using the Illumina Methylation 450K BeadChip annotation file, resulting in a total of 2032 uniquely mapped genes. We found 330 shared genes from our list of 2036 unique genes linked to our 2552 CpGs with FDR < 0.05. Different from CpG-level replication, the gene-level replication led to a replication rate of over 16%. Using the hypergeometric test, we estimated the probability of having 330 or more shared genes between the two EWASs if randomly sampling 2032 genes from the pool of 21,244 proximal genes of the 450K chip. The probability was estimated as *p* = 1.64e−23, meaning that it is highly unlikely to observe the 330 overlapping genes by chance.

In summary, this genome-wide DNA methylation profiling on the older-age Lothian Birth Cohorts identified 2, 552 CpG associated with mortality. In comparison with EWAS results on aging from the same sample, most mortality-associated CpGs do not display age-dependent patterns and the majority of age-associated CpGs do not correlate with mortality, but the limited overlap between them is highly significant from random. Besides, the predominately beneficial effects to survival from CpGs showing age-dependent methylation patterns could perhaps reflect active response to aging or represent an epigenetic regulation for successful aging.

## Additional files

Additional file 1: **Figure S1** PCA performed to identify batch effect, and ComBat correction using the *SVA* R-package with distributions of M-values before and after ComBat correction. (DOCX 571 kb)

Additional file 2: **Table S1** List of significant CpGs (FDR < 0.05) found using the Cox-proportional hazard model for finding CpGs associated with all-cause mortality. (CSV 427 kb)

Additional file 3: **Figure S2** QQ-plot for the EWAS on mortality in the combined dataset of LBC1921 and LBC1936. (DOCX 20 kb)

Additional file 4: **Table S3** List of pathways enriched by positive mortality association CpGs with FDR < 0.05. (XLSX 37 kb)

Additional file 5: **Table S2** List of pathways enriched by negative mortality association CpGs with FDR < 0.05. (XLSX 40 kb)

## Abbreviations

DNAm: DNA methylation
ECM: Extracellular matrix
EWAS: Epigenome-wide association study
FDR: False discovery rate
FWER: Family-wise error rate
GSEA: Gene Set Enrichment Analysis
LBC: Lothian Birth Cohort
NK: Natural killer cell
PCA: Principal component analysis
QC: Quality control
QQ plot: Quantile-quantile plot
