# Supplementary figures and images for: DNA methylome profiling of all-cause mortality in comparison with age-associated methylation patterns
Source: Clin Epigenetics. 2019 Feb 8;11:23. doi: 10.1186/s13148-019-0622-4 (PMC6368749; doi:10.1186/s13148-019-0622-4)

The LBC cohorts before combat batch correction:


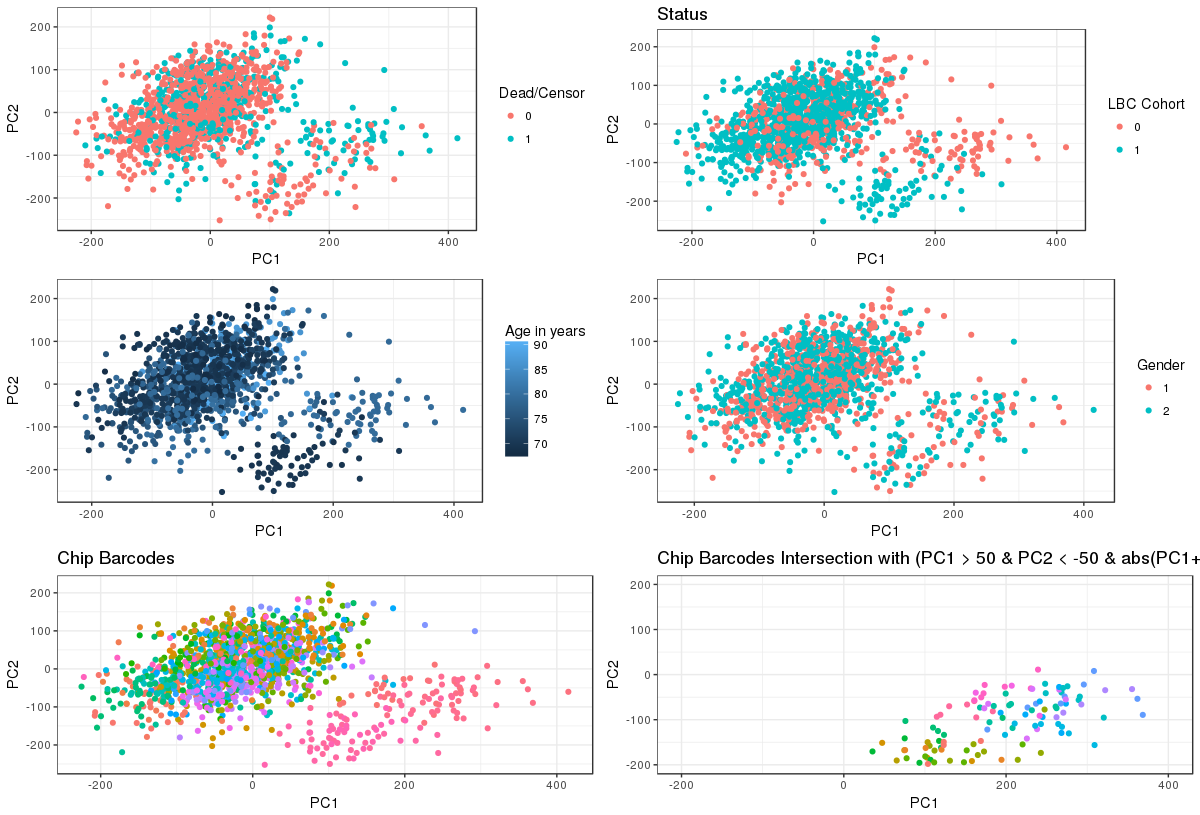


After correction:


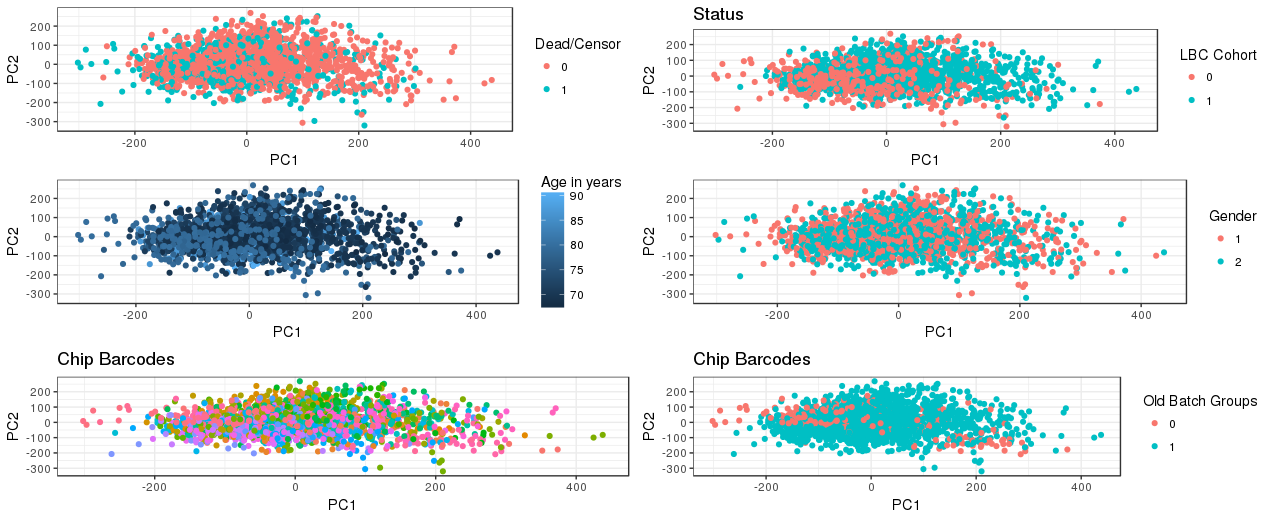

Supplement: Supplementary file 1 — Figure S1 PCA performed to identify batch effect, and ComBat correction using the SVA R-package with distributions of M-values before and after ComBat correction. (DOCX 571 kb) [file 13148_2019_622_MOESM1_ESM.docx]

**QQ plot for the combined LBC1921 and LBC1936 EWAS.**


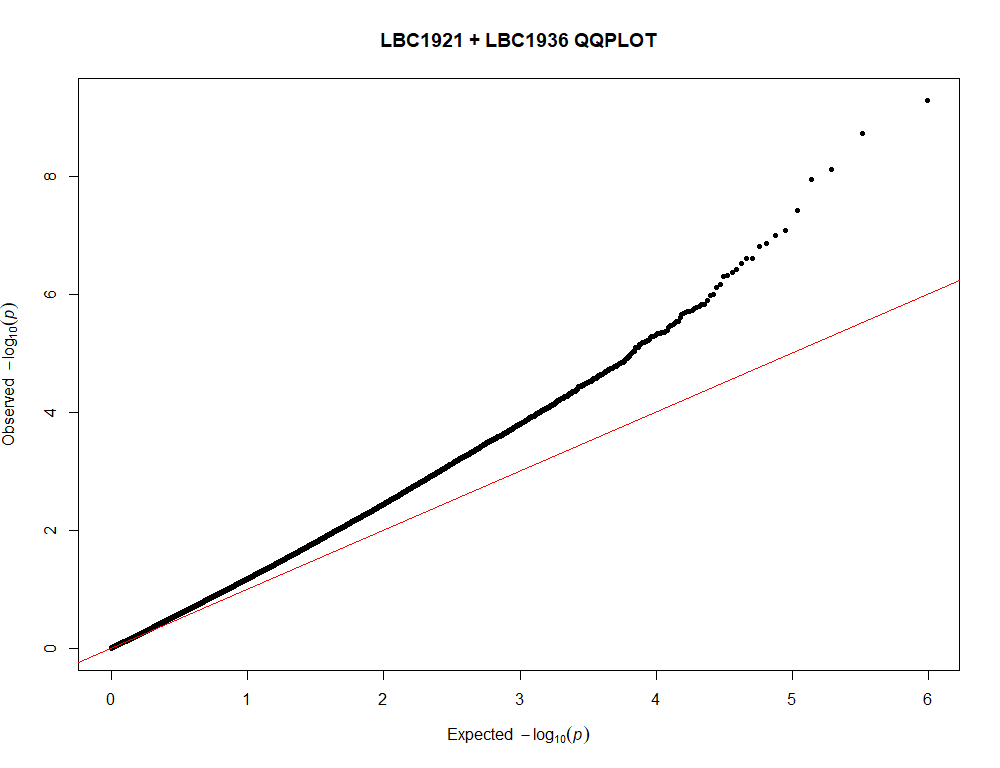

Supplement: Supplementary file 3 — Figure S2 QQ-plot for the EWAS on mortality in the combined dataset of LBC1921 and LBC1936. (DOCX 20 kb) [file 13148_2019_622_MOESM3_ESM.docx]
